# Distributed leadership as an enabler of community participation in primary health care in Malawi

**DOI:** 10.4102/phcfm.v18i1.5400

**Published:** 2026-08-18

**Authors:** Tony M. Majo, Martha K. Makwero

**Affiliations:** 1 Department of Family Medicine, School of Medicine and Oral Health, Kamuzu University of Health Sciences, Blantyre, Malawi

**Keywords:** primary health care, distributed leadership, community participation, health governance, Malawi

## Abstract

Community participation is a foundational principle of primary health care (PHC), yet its meaningful implementation remains limited in many low- and middle-income countries (LMICs), including Malawi. Although formal community governance structures such as Health Advisory Committees (HACs), Village Development Committees (VDCs) and Area Development Committees (ADCs) are recognised in policy, their influence on facility-level decision-making is often constrained by hierarchical leadership arrangements, weak coordination and persistent power asymmetries between health professionals and community representatives. This article examines community participation in Malawi’s PHC system through a distributed leadership (DL) lens and explores how leadership practices can be strengthened to enhance facility-community governance. A narrative review and policy analysis were conducted using National Health Policy documents and relevant literature on PHC governance, community participation and DL in Malawi. Community structures play an important role in representing community interests and monitoring service delivery but largely function in advisory capacities. Leadership authority remains concentrated at facility and district levels, limiting shared ownership. Coordination among health workers, community committees and local government structures is weak, resulting in fragmented leadership and limited accountability for service improvement. The article argues that challenges in community participation are fundamentally leadership challenges. Distributed leadership provides a practical framework for strengthening PHC by promoting shared responsibility, collaboration and accountability across community and facility levels.

## Introduction

Following the Declaration of Alma-Ata, primary health care (PHC) has increasingly been regarded not solely as a technical approach to health delivery but also as a social and political process based in the community.^1^ In low- and middle-income countries (LMICs), where health systems are operationally constrained by limited resources, the viability of PHC also depends to a marked extent on its approach to leadership.

Malawi has embraced PHC through decentralisation and the formation of community-based health structures aimed at developing community ownership and accountability.^2,3^ Yet, despite the conducive environment, community involvement in PHC remains disparate, typically in the form of support and consultancy. The leadership structure in PHC is mostly hierarchical, with decisions made at the facility or district level. This article argues that the challenges are leadership rather than participation issues in PHC and that distributed leadership (DL) is appropriate for improving community involvement in PHC in Malawi.

## Narrative review approach

This article used a narrative review and policy analysis approach to examine community participation and leadership in Malawi’s PHC system. Literature was sourced from PubMed, Scopus, Google Scholar, Web of Science, grey literature and policy documents from the Malawi Ministry of Health and World Health Organization (WHO). Search terms included ‘Primary Health Care’, ‘community participation’, ‘distributed leadership’, ‘health governance’, ‘Health Surveillance Assistants’ and ‘Malawi’. Included sources comprised peer-reviewed articles, policy documents, strategic reports and technical literature relevant to PHC governance, leadership and community health systems in Malawi and other LMICs. Data analysis was guided by Distributed Leadership Theory, particularly Spillane’s model, which informed examination of leadership relationships, collaborative decision-making and governance processes across Malawi’s PHC system.

## Distributed leadership as a health systems governance lens

Distributed leadership thinks about ‘leadership’ not as the characteristic of one individual but as ‘networked activity that emerges from interactions between plural actors’.^4^ ‘It is “stretched out” over actors, roles and contexts to be determined by relationships, routinisation and collective problem-solving rather than formal role possession’.^4,5^ Distributed leadership can be defined in many ways, most of which have common elements, such as shared, participatory and democratic leadership. However, this article adopts a definition of DL as a paradigm that moves the focus from individual leaders to collective practice by emphasising collaboration, shared responsibility, and the interactive relationship between leaders, followers and their environment.^2^

In the health system, DL is generally viewed as a collective phenomenon emerging from the interactions among multiple actors, including policymakers, managers, health practitioners and community members, as they collaborate, solve problems and make decisions within complex health environments.^6^ This leadership approach is particularly relevant in PHC, where service delivery depends on coordination across different levels and stakeholders. Evidence suggests that DL can strengthen PHC management capacity, enhance community engagement, support integrated and people-centred care, improve teamwork, and facilitate timely decision-making, particularly in resource-constrained settings such as LMICs. By promoting shared responsibility, collaborative problem-solving and local ownership, DL can improve the responsiveness and resilience of PHC systems. These potential benefits make the concept highly relevant to Malawi’s PHC system, where health service delivery operates within a complex context characterised by workforce shortages, resource limitations and the need for stronger coordination across community, facility and district levels.

According to the WHO, PHC can be defined as an approach to health and well-being of the whole society based on the preferences and needs of the individual, family and community. In PHC, health services are delivered in response to people’s needs and are expected to be timely, appropriate, accessible, relevant, equitable and effective. It is from this situation that the applicability of DL is derived. In PHC, leadership is required at the community, facility and district levels. Effective service delivery, outreach activities, resource utilisation and accountability depend on strong coordination among multiple actors, including health professionals, community members, volunteers and government representatives. In such a context, hierarchical management approaches may be less effective, as they can limit learning, responsiveness, participation and local ownership. Instead, PHC systems benefit from approaches that promote shared understanding, collective responsibility and the development of leadership capacity across all levels of the system. These principles strongly align with the concept of shared leadership, which emphasises collaboration, distributed decision-making and mutual accountability.

## Community participation and leadership arrangements in Malawi’s primary health care system

Malawi’s PHC system is delivered primarily through government and Christian Health Association of Malawi (CHAM) facilities, supported by Health Surveillance Assistants (HSAs) and multiple community governance structures. Community participation is embedded in national strategies and operationalised through Village Health Committees (VHCs), Health Advisory Committees (HACs) and local government development committees.^3^

However, participation in PHC is often procedural rather than substantive. Communities are frequently mobilised to support service delivery but rarely participate meaningfully in decision-making, priority setting or performance review.^7^ From a DL perspective, this reflects weak horizontal leadership arrangements and limited power-sharing within PHC governance structures. Although community structures exist, leadership is not genuinely distributed across them. More broadly, DL has been associated with improved teamwork, innovation and organisational efficiency. Within PHC systems, DL can strengthen management capacity, enhance community engagement, support integrated service delivery, improve teamwork and facilitate decision-making in resource-constrained settings such as LMICs, including Malawi.^8^ Even though DL is considered an effective strategy to deliver PHC services for low-income countries, evidence suggests that resource limitations may adversely affect the social and political dimensions of PHC. Common barriers cited include limited operational capacity of community health workers (CHWs) and community members to engage meaningfully in participatory processes, long travel distances, inadequate transport systems and weak participatory governance structures. As of 2025, Malawi has a gross domestic product (GDP) per capita of approximately US$600.00 – $700.00.^9^ In contrast, Botswana and South Africa record substantially higher GDP per capita levels of approximately $7660.00 – $8500.00 and $5970.00 – $6500.00, respectively. Although Botswana and South Africa are also classified as LMICs, their comparatively stronger economic bases have enabled greater investment in the health sector, which has supported more robust governance structures and more developed decentralised health management functions relative to Malawi.

## Leadership functions across community health structures

Health Surveillance Assistants play a central role in Malawi’s PHC system, delivering promotive, preventive and selected curative services at community level.^10^ Their proximity to households positions them as natural leaders within communities. Yet HSAs operate within tightly controlled, task-oriented systems shaped by vertical programmes and centralised supervision.^11^ Leadership is defined primarily as compliance with directives rather than collective problem-solving. As a result, HSAs’ leadership potential is constrained, and opportunities for shared leadership with communities are underutilised.

Village Health Committees are intended to mobilise communities, promote healthy behaviours and support HSAs. In theory, they provide a platform for DL at village level. In practice, many VHCs lack clear mandates, training and institutional support, limiting their influence.^12^ Their role is often confined to mobilisation, with minimal involvement in planning or monitoring. This marginalisation reflects leadership arrangements that do not value or enable shared authority.

Health Action Committees are designed to institutionalise community leadership at facility level by representing community interests and monitoring service delivery.^13^ However, power asymmetries between professional staff and community representatives persist, and HACs often function in an advisory rather than decision-making capacity.^14^ From a DL perspective, this indicates weak relational leadership and limited integration of community leadership into facility governance.

Local government structures such as Village Development Committees (VDCs) and Area Development Committees (ADCs) provide an opportunity for intersectoral leadership linking health with broader development priorities.^15^ Yet coordination between these structures and health facilities remains limited, reinforcing sectoral silos and fragmented leadership.

## Gaps in primary health care requiring distributed leadership

Viewed through a DL lens, several systemic gaps emerge. Leadership authority remains concentrated at facility and district levels, limiting shared ownership. Coordination among HSAs, community committees and facilities is weak, resulting in parallel rather than collective action.^11^ Community structures lack leadership capacity and institutional legitimacy, reducing their influence.^12^ Accountability mechanisms exist but are weakly linked to decision-making and service improvement.^14^

These gaps cannot be adequately addressed by technical reforms alone. They require deliberate redesign of leadership practices to enable shared decision-making, mutual accountability and collective learning.

## Strengthening distributed leadership in Malawi’s primary health care: A practice-oriented framework

To operationalise DL in Malawi’s PHC system, this article proposes strengthening DL using Spillane’s Distributed Leadership Framework, adapted to PHC governance.^5^ This framework emphasises three interacting elements: Leaders, followers and situations.

Firstly, leadership must be expanded beyond formal roles. Facility in-charges, HSAs, HAC members, VHCs and local leaders should be recognised as legitimate leaders within their domains. This requires deliberate role clarification and leadership development for community actors, not only professional staff.

Secondly, leadership practice must be embedded in routine interactions. Facility meetings, outreach planning, community review forums and monitoring processes should be redesigned as shared leadership spaces where decisions are jointly shaped. Health Surveillance Assistants and community representatives should participate meaningfully in facility planning and performance reviews.

Thirdly, the situation or context must support leadership distribution. This includes aligning policies, supervision systems and resource flows to reward collaboration rather than compliance. Supportive supervision, shared performance indicators, and feedback loops between communities and facilities are critical enablers. By focusing on leadership as practice rather than position, this framework offers a realistic pathway for strengthening DL within Malawi’s existing PHC structures.

## Policy alignment and implementation challenges

Malawi’s policy environment is broadly supportive of DL. The National Health Policy, Health Sector Strategic Plan and National Community Health Strategy emphasise decentralisation, community participation and accountability.^3^ These policies create formal space for shared leadership. However, implementation remains constrained by centralised control of resources, fragmented accountability systems and limited investment in leadership capacity at the community level.^11,14^ Without deliberate efforts to translate policy commitments into leadership practice, DL risks remain rhetorical rather than transformative.

## Conclusion

This article has argued that challenges to community participation in Malawi’s PHC system are fundamentally leadership issues. While community structures exist and policy frameworks are supportive, leadership remains hierarchical and insufficiently distributed. Distributed leadership offers a theoretically robust and contextually appropriate framework for strengthening PHC by enabling shared responsibility, collaboration and accountability across community and facility levels. Strengthening DL in Malawi requires moving beyond symbolic participation towards collective leadership practice. By investing in leadership capacity, redesigning governance routines, and aligning policies with collaborative practice, Malawi can enhance community ownership, improve PHC responsiveness and advance the original vision of PHC as articulated at Alma-Ata.
